# Tobacco use and harm perceptions among Appalachian youth

**DOI:** 10.1016/j.pmedr.2020.101089

**Published:** 2020-04-08

**Authors:** Delvon T. Mattingly, Lindsay K. Tompkins, Jayesh Rai, Clara G. Sears, Kandi L. Walker, Joy L. Hart

**Affiliations:** a University of Louisville, Department of Communication and Christina Lee Brown Envirome Institute, Louisville, KY, USA; b American Heart Association Tobacco Regulation and Addiction Center, Dallas, TX, USA

**Keywords:** Adolescent, Appalachian region, Cigarettes, Electronic cigarettes, Tobacco use, Tobacco products

## Abstract

Appalachian youth tobacco use rates exceed the national average. Additional inquiry is needed to better understand youth product perceptions and use patterns. This study examined tobacco harm perceptions and their relationship with tobacco use among Appalachian youth. From 2014 to 2016, a survey of Appalachian middle and high school students (N = 1,136) was conducted. Tobacco harm perceptions were assessed by tobacco use status, categorized as never user, cigarette only user, smokeless only user, e-cigarette only user, or polytobacco user. Descriptive characteristics were compared by tobacco use status and harm perceptions. Adjusted multivariable logistic regression models assessed the relationship between tobacco use status and harm perceptions. Over one-third of participants were tobacco users (34.6%; 4.7% cigarette only users, 3.3% smokeless only users, 5.6% e-cigarette only users, and 21.0% polytobacco users). Approximately half agreed that e-cigarettes cause health problems (54.4%), and 64.7% agreed that e-cigarettes are addictive. Most participants (83.4–92.3%) agreed that smoking and smokeless tobacco cause health problems and are addictive. Tobacco users more often disagreed that tobacco products cause health problems than did never users. Compared to never users, e-cigarette only users were more likely to disagree that smoking (AOR: 2.99, 95% CI: 1.30–6.90) and e-cigarettes cause health problems (AOR: 2.79, 95% CI: 1.64–4.75) and that e-cigarettes cause addiction (AOR: 2.48, 95% CI: 1.48–4.16). Most youth were aware of health dangers associated with smoking, but perceptions were split on whether e-cigarettes were associated with health problems or addiction.

The findings indicate the need for additional youth tobacco use prevention efforts.

## Highlights

• Over one-third had tried tobacco and over one-fifth had tried two or more products.
• Most participants recognized harms associated with conventional tobacco use.
• E-cigarettes were perceived as less harmful than conventional tobacco products.
• Smokers and polytobacco users had lower odds of indicating tobacco health risks.
• E-cigarette users were less likely to perceive e-cigarettes as harmful or addictive.

## 1 Introduction

More preventable diseases and deaths result from tobacco use in the United States (U.S.) than any other cause. Further, because most long-term tobacco users begin early in life, youth are especially vulnerable (U.S. Department of Health and Human Services, 2012, U.S. Department of Health and Human Services, 2014). For example, 90% of smokers tried their first cigarette prior to turning 18 (U.S. Department of Health and Human Services, 2012, U.S. Department of Health and Human Services, 2014). Because they have a lifetime of potential tobacco purchasing, youth are often targeted by tobacco company marketing and advertising (Marynak et al., 2018, Perks et al., 2018, US Department, 2016), despite restrictions limiting such communication. Thus, given that most tobacco use patterns begin in adolescence and tobacco companies target young people, considerable concern exists in the U.S. surrounding preventing youth tobacco uptake and reducing use in youth who consume tobacco products.

Despite these concerns, a number of U.S. youth use tobacco. Slightly>7% of middle school (7.2%) and 20% of high school (20.2%) students were considered current tobacco users (i.e., used any type of tobacco product in the past month) in 2016 (Jamal et al., 2017). Between 2011 and 2016, declines in U.S. middle and high school students’ current conventional cigarette smoking, from 4.3% to 2.2% for middle schoolers and 15.8% to 8% for high schoolers, were reported (Jamal et al., 2017, Arrazola et al., 2013). In this same time period, increases in U.S. middle and high school students’ current e-cigarette use, from 0.6% to 4.3% for middle schoolers and 1.5% to 11.3% for high schoolers, were reported (Jamal et al., 2017, Arrazola et al., 2013). Further, between 2015 and 2016, both current conventional cigarette smoking and use of e-cigarettes declined for high school students and use of e-cigarettes declined for middle school students (Jamal et al., 2017); however, for at least the past 5 years, the most frequently used tobacco product by middle and high school students has been e-cigarettes (Jamal et al., 2017). In spite of decreases in some areas of youth tobacco use, assessing the overall pattern of use in this five-year period revealed no significant change; that is, decreases in some areas (e.g., cigarettes, cigars, pipes, and smokeless tobacco) were offset by increases in use of newer tobacco products (e.g., e-cigarettes and hookah) by youth (Jamal et al., 2017). More recently, however, there has been a surge in e-cigarette use by these age groups, with use in 2017–2018 increasing by 77.8% among students in high school and 48.5% among students in middle school (Cullen et al., 2018). For example, in 2018, over one-fourth (27.1%) of high school students reported currently using at least one tobacco product (Gentzke et al., 2019). Of these students, over 40% used two or more tobacco products (Gentzke et al., 2019). Thus, previous strides in reducing overall tobacco product use as well as e-cigarette use have been eliminated (Cullen et al., 2018, Gentzke et al., 2019).

Tobacco use among youth is also higher in particular demographic groups in the United States, putting some youth more at risk for the long-term toll of tobacco consumption. For example, differences exist based on gender and race/ethnicity. One study found that male youth, from most racial/ethnic groups, were more likely to use tobacco than female youth, and that e-cigarettes were the most frequently used tobacco product for all racial/ethnic groups, other than Black youth who most frequently used cigars (Odani et al., 2018). Additionally, cigarette smoking rates are higher in people living in poverty (U.S. Department of Health and Human Services, 2014, Jamal et al., 2015). Thus, perhaps it is not surprising that the 13-state Appalachian region ranks high in both poverty and use of tobacco (Centers for Disease Control and Prevention, 2017); for example, the national smoking rate is 16.3%--19.8% in Appalachia and 16.0% in the Non-Appalachian areas (Appalachian Regional Commission, 2019). Beyond low socioeconomic status, several factors associated with life in Appalachia are believed to contribute to youth tobacco use. These factors include rural lifestyle, social acceptance of tobacco products, and family influence (Moreland et al., 2013, Curtis et al., 2010, Lutfiyya et al., 2008, West Virginia Department of Health and Human Resources, 1995, Meyer et al., 2008, Ahijevych et al., 2003, Rodriguez et al., 2016). However, little is known about how youth in the region view the relative harms of particular tobacco products, especially conventional cigarettes and e-cigarettes, the two most commonly used products by youth.

Several studies have found that youth perceive e-cigarettes as generally safe with no or minimal health consequences and that few youth indicate that using these devices is harmful or exposes one to toxic chemicals (Anand et al., 2015, Greenhill et al., 2016, Pepper and Brewer, 2014). Further, some studies have suggested that youth perceive e-cigarettes as less harmful than conventional tobacco products, which may influence initiation and use (Amrock et al., 2015, Tan et al., 2016, Ambrose et al., 2014, Pepper et al., 2016, Halpern-Felsher et al., 2004). However, research with vulnerable populations is limited (Stein et al., 2015, Ashford et al., 2016, Smiley et al., 2018), and even more so in youth populations (Kaleta et al., 2016, Thrasher et al., 2017). Furthermore, few studies have examined the use of e-cigarettes in Appalachian youth (Owusu et al., 2017). Many factors drive the youth tobacco use disparity in Appalachia, whose outcomes are well known—increased morbidity and mortality. Understanding how Appalachian youth perceive tobacco products may aid in designing targeted anti-tobacco campaigns (Thrasher et al., 2017), needed to address this public health crisis. This study examined tobacco harm perceptions and their relationship with tobacco use among Appalachian youth.

## 2 Methods

### 2.1 Data collection

The Youth Appalachian Tobacco Study (YATS) was conducted between fall 2014 and spring 2016. Questionnaires were distributed to middle and high school students in the Appalachian regions of three states: Kentucky, North Carolina, and New York. These states were selected based on their youth tobacco use rates: Kentucky ranked among the highest (17.9%), New York among the lowest (10.6%); and North Carolina was moderate (15.0%) (Centers for Disease Control and Prevention, 2014). Within the Appalachian regions of the three states, counties were selected by poverty levels that exceeded the national average (15.5%) and their state’s average (Kentucky 19.0%, North Carolina 17.2%, and New York 16.0%) (U.S. Census Bureau, 2014). Invitations to participate in the study were extended to public middle and high schools in each selected county, and if a school declined to participate, another school in the same or a nearby county was then invited. Students in ten schools in eight counties participated in the study. In Kentucky, schools in three counties participated (i.e., one school in each county). In North Carolina, schools in three counties participated (i.e., two schools in two counties and one school in another). In New York, schools in two counties participated (i.e., one school in each county).

The investigator-generated questionnaire included items on tobacco-related perceptions, attitudes, and use behaviors as well as demographic characteristics. Questions regarding views of tobacco harms, marketing and advertising, and use (friends, family, and self) were asked. Prior to data collection, parents or guardians received a letter (from the investigators and distributed by school administrators) describing the study and giving them the option to decline their child’s participation. Students were also given assent forms and could elect not to participate in the study. On average, the questionnaire took 40 minutes to complete, and data collection took place during the regular school day. The University of Louisville’s Institutional Review Board approved this research.

### 2.2 Participants

A total of 1,280 students participated in YATS. Participants who did not respond to all of the harm perception questions (n = 32) were excluded from the analysis, as were participants missing tobacco use information (n = 94). Participants missing information on variables used for adjustment in logistic regression models were also excluded from the analysis (n = 18). This resulted in a final sample size of 1,136.

### 2.3 Measures

Demographic characteristics such as age, gender, race/ethnicity, school type (middle or high school), and state (Kentucky, North Carolina, or New York) were included in the analysis.

#### 2.3.1 Tobacco use status

Participants were asked about former and current (i.e., past 30-day) cigarette, smokeless tobacco, and e-cigarette use. Participants who were former or current exclusive cigarette users were classified as cigarette only users, participants who were former or current exclusive smokeless tobacco users were classified as smokeless only users, participants who were former or current exclusive e-cigarette users were classified as e-cigarette only users, participants who were former or current users of two or more of the previous three tobacco products were classified as polytobacco users, and participants who had never used cigarettes, smokeless tobacco, or e-cigarettes were classified as never users.

#### 2.3.2 Tobacco harm perceptions

Participants indicated whether they agreed that: smoking causes health problems, addiction, or difficulty quitting; smokeless tobacco causes health problems, addiction, or difficulty quitting; and e-cigarettes cause health problems, addiction, or difficulty quitting. Participants responded indicating their level of agreement with each statement via a five-point Likert Scale (1 = Strongly Disagree; 2 = Disagree; 3 = Neither Disagree nor Agree; 4 = Agree; 5 = Strongly Agree). Agreement statements were dichotomized (i.e., disagree, responses 1–3; agree, responses 4–5). For each tobacco product, addiction and difficulty quitting were combined, and participants who responded with 4 or 5 (i.e., Agree or Strongly Agree) to at least one of the statements were categorized as “agree”.

### 2.4 Statistical analysis

Frequencies and percentages of categorical variables and means (SD) and medians (min–max) of continuous variables were computed and stratified by tobacco use status. Comparisons between descriptive characteristics and harm perceptions by tobacco use status were calculated by chi-square tests of independence for categorical variables and analysis of variance (ANOVA) for continuous variables. Further tests were conducted to compare never users to tobacco users (i.e., cigarette only users, smokeless only users, e-cigarette only users, or polytobacco users) by descriptive characteristics and harm perceptions using chi-square tests of independence or Student’s t-tests, as appropriate. Distributions of descriptive characteristics and tobacco use status by harm perceptions were examined. Differences in these distributions were evaluated using chi-square tests of independence or Fisher’s exact test, as appropriate, for categorical variables and Student’s t-tests for continuous variables.

A concordance analysis was employed to evaluate agreement comparisons between each harm statement. P-values were calculated using McNemar’s test.

Multivariable logistic regression models assessed the relationship between tobacco use status and tobacco harm statement agreement (i.e., six regression models were conducted, one for each harm perception statement), with never users as the referent group and disagreeing with harm statements as the outcome, after adjustment for gender, school type, and state. Adjusted odds ratios (AORs) and 95% confidence intervals are reported. Data were analyzed using SAS 9.4 (Cary, N.C.).

## 3 Results

### 3.1 Participant characteristics by tobacco use status

Table 1 presents participant characteristics by tobacco use status. Of the total sample, 65.4% were never users (n = 743), 4.7% were cigarette only users (n = 53), 3.3% were smokeless only users (n = 38), 5.6% were e-cigarette only users (n = 64), and 21.0% were polytobacco users (n = 238). For product former and current use, the findings were as follows: cigarettes 14.3% and 10.2%, respectively; e-cigarettes 14.4% and 9.3%, respectively; smokeless 9.0% and 9.1%, respectively. The majority agreed that “Smoking causes health problems” (92.3%) and that “Smoking is addictive/difficult to quit” (91.7%). Most participants (83.4%) agreed that smokeless tobacco causes health problems and is addictive or difficult to quit. In contrast, just over half (54.4%) thought e-cigarettes caused health problems and almost two-thirds (64.7%) thought that they were addictive or difficult to quit.

**Table 1:** Participant Characteristics by Tobacco Use Status (*N* = 1136).

Characteristics	Total *N* (%)	Tobacco Use Status *n* (%)					*P*
		Never	Cigarette	Smokeless	E-cigarette	Polytobacco	
	(*N* = 1136)	(*n* = 743)	(*n* = 53)	(*n* = 38)	(*n* = 64)	(*n* = 238)	
Gender							
Male	573 (50.4)	359 (48.3)	21 (39.6)	30 (79.0)	26 (40.6)	137 (57.6)	*<0.001^a^*
Female	563 (49.6)	384 (51.7)	32 (60.4)	8 (21.0)	38 (59.4)	101 (42.4)	*0.049^b^*
Race/Ethnicity							
White/Caucasian	973 (88.4)	637 (87.7)	44 (89.8)	35 (94.6)	54 (87.1)	203 (89.8)	0.67^a^
Non-White/Caucasian	127 (11.6)	89 (12.3)	5 (10.2)	2 (5.4)	8 (12.9)	23 (10.2)	0.30^b^
Age							
Mean (SD)	13.7 ± 1.9	13.3 ± 1.7	14.3 ± 2.0	14.5 ± 2.1	14.0 ± 1.8	14.8 ± 1.8	*<0.001^a^*
Median (min–max)	14 (11–19)	13 (11–19)	14 (11–19)	14 (12–19)	14 (11–18)	15 (11–19)	*<0.001^b^*
School type							
Middle school	695 (61.2)	513 (69.0)	30 (56.6)	20 (52.6)	35 (54.7)	97 (40.8)	*<0.001^a^*
High school	441 (38.8)	230 (31.0)	23 (43.4)	18 (47.4)	29 (45.3)	141 (59.2)	*<0.001^b^*
School state							
Kentucky	399 (35.1)	217 (29.2)	23 (43.4)	21 (55.3)	21 (32.8)	117 (49.2)	*<0.001^a^*
North Carolina	525 (46.2)	370 (49.8)	22 (41.5)	6 (15.8)	34 (53.1)	93 (39.1)	*<0.001^b^*
New York	212 (18.7)	156 (21.0)	8 (15.1)	11 (28.9)	9 (14.1)	28 (11.8)	
Cigarette use history							
Former use	163 (14.3)	–	41 (77.4)	–	–	122 (51.3)	*0.002*
Current use	116 (10.2)	–	12 (22.6)	–	–	104 (43.7)	
Smokeless use history							
Former use	102 (9.0)	–	–	24 (63.2)	–	78 (32.8)	0.067
Current use	103 (9.1)	–	–	14 (36.8)	–	89 (37.4)	
E-cigarette use history							
Former use	164 (14.4)	–	–	–	44 (68.8)	120 (50.4)	0.13
Current use	106 (9.3)	–	–	–	20 (31.2)	86 (36.1)	
***Harm Perceptions***							
Smoking causes health problems							
Agree	1049 (92.3)	705 (94.9)	44 (83.0)	33 (86.8)	56 (87.5)	211 (88.7)	*<0.001^a^*
Disagree	87 (7.7)	38 (5.1)	9 (17.0)	5 (13.2)	8 (12.5)	27 (11.3)	*<0.001^b^*
Smokeless tobacco causes health problems							
Agree	947 (83.4)	646 (86.9)	39 (73.6)	31 (81.6)	52 (81.3)	179 (75.2)	*<0.001^a^*
Disagree	189 (16.6)	97 (13.1)	14 (26.4)	7 (18.4)	12 (18.7)	59 (24.8)	*<0.001^b^*
E-cigarettes cause health problems							
Agree	618 (54.4)	458 (61.6)	22 (41.5)	24 (63.2)	24 (37.5)	90 (37.8)	*<0.001^a^*
Disagree	518 (45.6)	285 (38.4)	31 (58.5)	14 (36.8)	40 (62.5)	148 (62.2)	*<0.001^b^*
Smoking is addictive/difficult to quit							
Agree	1042 (91.7)	692 (93.1)	44 (83.0)	34 (89.5)	59 (92.2)	213 (89.5)	0.059^a^
Disagree	94 (8.3)	51 (6.9)	9 (17.0)	4 (10.5)	5 (7.8)	25 (10.5)	*0.018^b^*
Smokeless tobacco is addictive/difficult to quit							
Agree	947 (83.4)	633 (85.2)	42 (79.3)	30 (79.0)	55 (85.9)	187 (78.6)	0.12^a^
Disagree	189 (16.6)	110 (14.8)	11 (20.7)	8 (21.0)	9 (14.1)	51 (21.4)	*0.023^b^*
E-cigarettes are addictive/difficult to quit							
Agree	735 (64.7)	518 (69.7)	31 (58.5)	26 (68.4)	31 (48.4)	129 (54.2)	*<0.001^a^*
Disagree	401 (35.3)	225 (30.3)	22 (41.5)	12 (31.6)	33 (51.6)	109 (45.8)	*<0.001^b^*

a: Chi-square (categorical) or ANOVA (continuous) p-values comparing all tobacco use status groups by presented characteristics and harm perceptions.

b: Chi-square (categorical) or Student's *t*-test (continuous) p-values comparing never users to ever tobacco users by presented characteristics and harm perceptions.

Missing values: Race/Ethnicity (n = 36).

For descriptive characteristics, the distribution of tobacco use status differed by gender (p < 0.001), age (p < 0.001), school type (p < 0.001), school state (p < 0.001), and cigarette use history (p = 0.002). Comparisons between never users and tobacco users significantly differed by gender (p = 0.049), age (p < 0.001), school type (p < 0.001), and school state (p < 0.001). Harm perceptions “Smoking causes health problems”, “Smokeless tobacco causes health problems”, “E-cigarettes cause health problems”, and “E-cigarettes are addictive/difficult to quit” significantly differed by tobacco use status and in comparisons between never users and tobacco users (p < 0.001 for all). Comparisons between never users and tobacco users by agreement with “Smoking is addictive/difficult to quit” and “Smokeless tobacco is addictive/difficult to quit” significantly differed (p = 0.018 and p = 0.023, respectively).

### 3.2 Participant characteristics by perceptions of tobacco product harms

Participant characteristics stratified by tobacco harm perceptions are presented in Table 2, Table 3.

**Table 2:** Participant Characteristics by Perceptions of Health Problems (*N* = 1136).

Characteristics	Total *N* (%)	Perceptions of Health Problems *N* (%)								
		Smoking causes health problems			Smokeless tobacco causes health problems			E-cigarettes cause health problems		
		Agree	Disagree	*P*^a^	Agree	Disagree	*P*^a^	Agree	Disagree	*P*^a^
Total *N* (%)		1049 (92.3)	87 (7.7)		947 (83.4)	189 (16.6)		618 (54.4)	518 (45.6)	
Gender				*0.003*			*0.013*			0.66
Male	573 (50.4)	516 (90.1)	57 (9.9)		462 (80.6)	111 (19.4)		308 (53.8)	265 (46.2)	
Female	563 (49.6)	533 (94.7)	30 (5.3)		485 (86.2)	78 (13.8)		310 (55.1)	253 (44.9)	
Race/Ethnicity				0.49			0.24			*0.044*
White/Caucasian	973 (88.4)	905 (93.0)	68 (7.0)		821 (84.4)	152 (15.6)		544 (55.9)	429 (44.1)	
Non-White/Caucasian	127 (11.6)	116 (91.3)	11 (8.7)		102 (80.3)	25 (19.7)		59 (46.5)	68 (53.5)	
Age				0.42			0.55			*0.024*
Mean (SD)	13.7 ± 1.9	13.8 ± 1.9	13.6 ± 1.5		13.7 ± 1.9	13.8 ± 1.8		13.6 ± 1.9	13.9 ± 1.9	
Median (min–max)	14 (11–19)	14 (11–19)	14 (11–18)		13 (11–19)	14 (11–19)		13 (11–19)	14 (11–19)	
School type				*0.014*			0.13			0.63
Middle school	695 (61.2)	631 (90.8)	64 (9.2)		570 (82.0)	125 (18.0)		382 (55.0)	313 (45.0)	
High school	441 (38.8)	418 (94.8)	23 (5.2)		377 (85.5)	64 (14.5)		236 (53.5)	205 (46.5)	
School state				*<0.001*			0.25			0.08
Kentucky	399 (35.1)	343 (86.0)	56 (14.0)		325 (81.5)	74 (18.5)		224 (56.1)	175 (43.9)	
North Carolina	525 (46.2)	503 (95.8)	22 (4.2)		448 (85.3)	77 (14.7)		268 (51.1)	257 (48.9)	
New York	212 (18.7)	203 (95.8)	9 (4.2)		174 (82.1)	38 (17.9)		126 (59.4)	86 (40.6)	
Tobacco use status^b^				*<0.001*			*<0.001*			*<0.001*
Never users	743 (65.4)	705 (94.9)	38 (5.1)		646 (86.9)	97 (13.1)		458 (61.6)	285 (38.4)	
Cigarette only users	53 (4.7)	44 (83.0)	9 (17.0)	*0.003*	39 (73.6)	14 (26.4)	*0.007*	22 (41.5)	31 (58.5)	*0.004*
Smokeless only users	38 (3.3)	33 (86.8)	5 (13.2)	0.051	31 (81.6)	7 (18.4)	0.34	24 (63.2)	14 (36.8)	0.85
E-cigarette only users	64 (5.6)	56 (87.5)	8 (12.5)	*0.023*	52 (81.3)	12 (18.7)	0.20	24 (37.5)	40 (62.5)	*<0.001*
Polytobacco users	238 (21.0)	211 (88.7)	27 (11.3)	*<0.001*	179 (75.2)	59 (24.8)	*<0.001*	90 (37.8)	148 (62.2)	*<0.001*

a: Chi-square or Fisher's Exact (categorical) or *t*-test (continuous) p-values comparing harm/addiction agreement by presented covariates.

b: The first p-value compares all tobacco use groups at once. Additional p-values individually compare each corresponding tobacco group to never users.

Missing values: Race/Ethnicity (n = 36).

**Table 3:** Participant Characteristics by Perceptions of Addiction (*N* = 1136).

Characteristics	Total *N* (%)	Perceptions of Addiction *N* (%)								
		Smoking is addictive/difficult to quit			Smokeless tobacco is addictive/difficult to quit			E-cigarettes are addictive/ difficult to quit		
		Agree	Disagree	*P*^a^	Agree	Disagree	*P*^a^	Agree	Disagree	*P*^a^
Total *N* (%)		1042 (91.7)	94 (8.3)		947 (83.4)	189 (16.6)		735 (64.7)	401 (35.3)	
Gender				*0.002*			*0.019*			0.28
Male	573 (50.4)	511 (89.2)	62 (10.8)		463 (80.8)	110 (19.2)		362 (63.2)	211 (36.8)	
Female	563 (49.6)	531 (94.3)	32 (5.7)		484 (86.0)	79 (14.0)		373 (66.3)	190 (33.7)	
Race/Ethnicity				0.09			*0.001*			*0.015*
White/Caucasian	973 (88.4)	900 (92.5)	73 (7.5)		825 (84.8)	148 (15.2)		643 (66.1)	330 (33.9)	
Non-White/Caucasian	127 (11.6)	112 (88.2)	15 (11.8)		94 (74.0)	33 (26.0)		70 (55.1)	57 (44.9)	
Age				0.86			0.26			0.059
Mean (SD)	13.7 ± 1.9	13.7 ± 1.9	13.8 ± 1.9		13.8 ± 1.9	13.6 ± 1.8		13.7 ± 1.9	13.9 ± 1.9	
Median (min–max)	14 (11–19)	14 (11–19)	14 (11–18)		14 (11–19)	13 (11–19)		13 (11–19)	14 (11–19)	
School type				0.44			*0.005*			0.67
Middle school	695 (61.2)	634 (91.2)	61 (8.8)		562 (80.9)	133 (19.1)		453 (65.2)	242 (34.8)	
High school	441 (38.8)	408 (92.5)	33 (7.5)		385 (87.3)	56 (12.7)		282 (64.0)	159 (36.0)	
School state				*0.007*			*0.002*			*0.004*
Kentucky	399 (35.1)	352 (88.2)	47 (11.8)		312 (78.2)	87 (21.8)		252 (63.2)	147 (36.8)	
North Carolina	525 (46.2)	492 (93.7)	33 (6.3)		450 (85.7)	75 (14.3)		325 (61.9)	200 (38.1)	
New York	212 (18.7)	198 (93.4)	14 (6.6)		185 (87.3)	27 (12.7)		158 (74.5)	54 (25.5)	
Tobacco use status^b^				0.059			0.12			*<0.001*
Never users	743 (65.4)	692 (93.1)	51 (6.9)		633 (85.2)	110 (14.8)		518 (69.7)	225 (30.3)	
Cigarette only users	53 (4.7)	44 (83.0)	9 (17.0)	*0.014*	42 (79.3)	11 (20.7)	0.24	31 (58.5)	22 (41.5)	0.09
Smokeless only users	38 (3.3)	34 (89.5)	4 (10.5)	0.33	30 (79.0)	8 (21.0)	0.29	26 (68.4)	12 (31.6)	0.87
E-cigarette only users	64 (5.6)	59 (92.2)	5 (7.8)	0.80	55 (85.9)	9 (14.1)	0.87	31 (48.4)	33 (51.6)	*<0.001*
Polytobacco users	238 (21.0)	213 (89.5)	25 (10.5)	0.068	187 (78.6)	51 (21.4)	*0.016*	129 (54.2)	109 (45.8)	*<0.001*

a: Chi-square or Fisher's Exact (categorical) or *t*-test (continuous) p-values comparing harm/addiction agreement by presented covariates.

b: The first p-value compares all tobacco use groups at once. Additional p-values individually compare each corresponding tobacco group to never users.

Missing values: Race/Ethnicity (n = 36).

Agreement with tobacco harm statements was consistently higher among female participants than male participants, though agreement only significantly differed by gender when smoking and smokeless tobacco harm statements were assessed.

Smoking (causes health problems: 94.8% vs. 90.8%, addictive: 92.5% vs. 91.2%) and smokeless tobacco (causes health problems: 85.5% vs. 82.0%, addictive: 87.3% vs. 80.9%) harm statement agreement was higher among high school participants than middle school participants, though only significantly so for smoking causes health problems (p = 0.014) and smokeless tobacco is addictive/difficult to quit (p = 0.005) statements.

Perceptions of tobacco product harm differed by state. Participants residing in Kentucky had the lowest agreement for smoking (causes health problems: 86.0%, p < 0.001; addictive: 88.2%, p = 0.007) and smokeless tobacco (causes health problems: 81.5%, p > 0.05; addictive: 78.2%, p = 0.002) harm statements, and participants residing in North Carolina had the lowest agreement for e-cigarette harm statements (causes health problems: 51.1%, p > 0.05; addictive: 61.9%, p = 0.004).

### 3.3 Concordance analysis

Comparing agreement by harm perceptions is reported in Table 4. When comparing “Smoking causes health problems” and “Smoking causes addiction”, there was evidence of concordance (p = 0.41) as well as when comparing “Smokeless tobacco causes health problems” and “Smokeless tobacco causes addiction” (p = 1.00). For the rest of the comparisons, there was significant evidence of discordance (p < 0.001).

**Table 4:** Concordance Analysis of Harm Perceptions (*N* = 1136).

	Harm Perceptions *N* (%)																	
	Smoking						Smokeless Tobacco						E-cigarettes					
	Causes Health Problems			Addictive			Causes Health Problems			Addictive			Cause Health Problems			Addictive		
Harm Perceptions	Agree	Disagree	*P*^a^	Agree	Disagree	*P*^a^	Agree	Disagree	*P*^a^	Agree	Disagree	*P*^a^	Agree	Disagree	*P*^a^	Agree	Disagree	*P*^a^
Smoking																		
Causes health problems						–			–			–			–			–
Agree	–	–		–	–		–	–		–	–		–	–		–	–	–
Disagree	–	–		–	–		–	–		–	–		–	–		–	–	–
Addictive			0.41			–			–			–			–			
Agree	1010 (88.9)	32 (2.8)		–	–		–	–		–	–		–	–		–	–	–
Disagree	39 (3.4)	55 (4.9)		–	–		–	–		–	–		–	–		–	–	–
Smokeless tobacco																		
Causes health problems			*<0.001*			*<0.001*			–			–			–			–
Agree	935 (82.3)	12 (1.1)		923 (81.2)	24 (2.1)		–	–		–	–		–	–		–	–	–
Disagree	114 (10.0)	75 (6.6)		119 (10.5)	70 (6.2)		–	–		–	–		–	–		–	–	–
Addictive			*<0.001*			*<0.001*			1.00			–			–			
Agree	920 (81.0)	27 (2.4)		925 (81.4)	22 (2.0)		861 (75.8)	86 (7.6)		–	–		–	–		–	–	–
Disagree	129 (11.3)	60 (5.3)		117 (10.3)	72 (6.3)		86 (7.6)	103 (9.0)		–	–		–	–		–	–	–
E-cigarettes																		
Cause health problems			*<0.001*			*<0.001*			*<0.001*			*<0.001*			–			–
Agree	608 (53.5)	10 (0.9)		603 (53.1)	15 (1.3)		581 (51.1)	37 (3.3)		576 (50.7)	42 (3.7)		–	–		–	–	–
Disagree	441 (38.8)	77 (6.8)		439 (38.6)	79 (7.0)		366 (32.2)	152 (13.4)		371 (32.7)	147 (12.9)		–	–		–	–	–
Addictive			*<0.001*			*<0.001*			*<0.001*			*<0.001*			*<0.001*			
Agree	709 (62.4)	26 (2.3)		718 (63.2)	17 (1.5)		661 (58.2)	74 (6.5)		689 (60.7)	46 (4.0)		540 (47.5)	195 (17.2)		–	–	–
Disagree	340 (29.9)	61 (5.4)		324 (28.5)	77 (6.8)		286 (25.2)	115 (10.1)		258 (22.7)	143 (12.6)		78 (6.9)	323 (28.4)		–	–	–

a: McNemar p-values evaluating agreement between harm perceptions.

### 3.4 Likelihood of disagreeing with tobacco harm statements

#### 3.4.1 Smoking harm statements

After adjusting for gender, school type, and state, when compared to never users, cigarette only users had a nearly four-fold increased likelihood of disagreeing with the statement “Smoking causes health problems” (AOR: 3.83; CI: 1.68, 8.74), whereas e-cigarette only users had a nearly three times increased likelihood (AOR: 2.99; CI: 1.30, 6.90) and polytobacco users had greater than two times increased likelihood (AOR: 2.22; CI: 1.28, 3.87) of disagreeing with the same statement (see Table 5). Cigarette only users were the only tobacco use group that had a significantly increased likelihood of disagreeing with the statement “Smoking causes addiction” when compared to never users (AOR: 2.88; CI: 1.31, 6.35).

**Table 5:** Multivariable Logistic Regression Models of Harm Perceptions by Tobacco Use Status (*N* = 1136).

Tobacco Use Status	Harm Perception Statements (Disagree versus Agree)			
	Smoking causes health problems		Smoking causes addiction	
	AOR (95% CI)^a^	*P*	AOR (95% CI)^a^	*P*
Never users	1.0 (Ref)		1.0 (Ref)	
Cigarette only users	**3.83 (1.68, 8.74)**	*0.001*	**2.88 (1.31, 6.35)**	*0.009*
Smokeless only users	2.03 (0.72, 5.78)	0.18	1.25 (0.42, 3.75)	0.68
E-cigarette only users	**2.99 (1.30, 6.90)**	*0.010*	1.23 (0.47, 3.23)	0.68
Polytobacco users	**2.22 (1.28, 3.87)**	*0.005*	1.47 (0.86, 2.51)	0.16


**Tobacco Use Status**	**SLT causes health problems^b^**		**SLT causes addiction^b^**	
	**AOR (95% CI)^a^**	***P***	**AOR (95% CI)^a^**	***P***

Never users	1.0 (Ref)		1.0 (Ref)	
Cigarette only users	**2.73 (1.41, 5.28)**	*0.003*	1.58 (0.78, 3.20)	0.21
Smokeless only users	1.48 (0.63, 3.51)	0.37	1.40 (0.61, 3.21)	0.43
E-cigarette only users	1.74 (0.89, 3.40)	0.11	1.02 (0.49, 2.15)	0.95
Polytobacco users	**2.55 (1.72, 3.77)**	*<0.001*	**1.66 (1.12, 2.47)**	*0.012*


**Tobacco Use Status**	**E-cigarettes cause health problems**		**E-cigarettes cause addiction**	

**AOR (95% CI)^a^**	***P***	**AOR (95% CI)^a^**	***P***

Never users	1.0 (Ref)		1.0 (Ref)	
Cigarette only users	**2.37 (1.34, 4.20)**	*0.003*	1.63 (0.92, 2.88)	0.10
Smokeless only users	0.97 (0.49, 1.91)	0.92	1.01 (0.50, 2.05)	0.98
E-cigarette only users	**2.79 (1.64, 4.75)**	*<0.001*	**2.48 (1.48, 4.16)**	*<0.001*
Polytobacco users	**2.84 (2.07, 3.90)**	*<0.001*	**1.89 (1.38, 2.59)**	*<0.001*

a: Adjusted odds ratios (AOR) and 95% confidence intervals adjusted for gender, school type, and state.

b: SLT: Smokeless tobacco.

#### 3.4.2 Smokeless tobacco harm statements

When compared to never users, cigarette only users (AOR: 2.73; CI: 1.41, 5.28) and polytobacco users (AOR: 2.55; CI: 1.72, 3.77) had greater odds of disagreeing that “Smokeless tobacco causes health problems” (see Table 5). For the harm statement “Smokeless tobacco causes addiction”, polytobacco users were the only tobacco use group that had an increased likelihood of disagreeing when compared to never users (AOR: 1.66; CI: 1.12, 2.47).

#### 3.4.3 E-Cigarette harm statements

For “E-cigarettes cause health problems”, compared to never users, three tobacco use groups had increased likelihood of disagreeing: cigarette only users (AOR: 2.37; CI: 1.34, 4.20), e-cigarette only users (AOR: 2.79; CI: 1.64, 4.75), and polytobacco users (AOR: 2.84; CI: 2.07, 3.90) (see Table 5). Furthermore, compared to never users, e-cigarette only users (AOR: 2.48; CI: 1.48, 4.16) and polytobacco users (AOR: 1.89; CI: 1.38, 2.59) had increased likelihoods of disagreeing with the statement “E-cigarettes cause addiction”.

## 4 Discussion

Nearly two-thirds (65.4%) of these youth participants reported never trying tobacco, despite cultural and regional histories as well as acceptance of tobacco (e.g., growing tobacco, families relying on tobacco sales for income, smoking in homes and public places) in many of these counties (Moreland et al., 2013, Lutfiyya et al., 2008, Meyer et al., 2008, Owusu et al., 2017, Owusu et al., 2019). However, slightly over one-third had tried one or more forms of tobacco, and over one-fifth had tried at least two forms; certainly, both of these findings are causes for concern. More specifically, about two-fifths of the youth (39.3%) who had ever tried or currently use e-cigarettes had done so in the past month, and approximately the same percentage (41.6%) who had ever tried or currently use conventional tobacco products had done so in the same timeframe. Because tobacco use patterns established in adolescence put individuals at greater risk of long-term use and the associated health risks, any level of former or current use by youth is a cause for concern (U.S. Department of Health and Human Services, 2012, U.S. Department of Health and Human Services, 2014, US Department, 2016). Thus, the frequency of current use (i.e., past 30-day) for both e-cigarettes and conventional tobacco suggests that these youth are at risk for continued consumption.

Causal links between conventional tobacco use and health problems were widely recognized (e.g., 92.3% indicated smoking causes health problems; 83.4% indicated smokeless tobacco causes health problems; most agreed that smoking and smokeless tobacco use cause addiction, 91.7% and 83.4%, respectively). Far fewer participants believed that e-cigarettes cause health problems (54.4%) and addiction (64.7%). These findings suggest the need for communication campaigns to raise awareness of the health dangers of e-cigarette use by youth, including the nicotine content of some e-cigarette products and the associated risks as well as the possibility of e-cigarette or vaping product use-associated lung injury (EVALI) (Siegel et al., 2019, Centers for Disease Control and Prevention, 2020).

Of the three states, Kentucky has the highest youth tobacco use rates (Centers for Disease Control and Prevention, 2014) and, for participants in this study, the lowest levels of agreement that smoking or using smokeless tobacco causes health problems or is addictive or difficult to quit. Participants from North Carolina were least convinced that using e-cigarettes is linked to health issues or addictive. These findings underscore the need for geographically-focused health campaigns for youth especially vulnerable to tobacco use. High school students were more likely than middle school students to agree that smoking causes health problems and that using smokeless tobacco is addictive.

Overall, these findings indicate that the Appalachian youth surveyed are generally aware of the health dangers associated with the use of conventional tobacco products and that they are less certain regarding potential health dangers of e-cigarette use. The varying perceptions on e-cigarette safety may reflect the relative newness of these products; outcomes of marketing efforts (Pokhrel et al., 2015); limited information on the health effects of e-cigarettes (Ratajczak et al., 2018), especially regarding long-term use; or scientific uncertainty surrounding the effects and value of these devices (e.g., cessation tool, harm reduction potential, new path of addiction) (Kalkhoran and Glantz, 2016), resulting in fewer health information campaigns. Further, views of harm appear to be shaped by tobacco use group; thus, warranting deeper investigation of these influences in future studies. Given that many youth use two or more tobacco products, research to better understand their motivations, use patterns, and harm perceptions is especially important, and one of this study’s contributions is addressing polytobacco users in an especially vulnerable youth population.

With these views, e-cigarette users may be more at risk of transitioning from e-cigarettes to conventional forms of tobacco (e.g., combustible cigarettes, smokeless tobacco) and less likely to heed any new cautions on newer tobacco product use. Some studies have found that youth who use e-cigarettes are more likely to become conventional tobacco users (National Academies of Sciences, Engineering, and Medicine, 2018, Soneji et al., 2017), and future research could further examine potential associations between harm perceptions and such transitions. In addition, our findings suggest that, although the Appalachian youth surveyed generally agreed that tobacco use causes health problems and addiction, the subset using tobacco products may be vulnerable to continued use as many dispute or question the harmful and/or addictive properties of these products.

Future anti-tobacco messaging could target such groups and clarify information on harms and potential harms as well as addiction. Because e-cigarette users were the most skeptical about e-cigarette harms, this use group is an important one to target in future health campaigns about new and emerging tobacco products. In particular, such campaigns could convey information about the FDA’s comprehensive policy on nicotine due to the associated health dangers of nicotine consumption. Certainly, more health messaging targeting vulnerable, rural youth is needed. Additionally, our study findings suggest that work remains in educating middle school students as well as students prior to middle school on the harms of tobacco product use. Overall, the findings of the current study reinforce the need for vigilance in restricting youth access to tobacco products and the need to increase health campaigns that clarify scientific findings on conventional tobacco products as well as uncertainty surrounding e-cigarette safety. In addition, the findings of this study emphasize the need to regulate e-cigarette marketing that targets youth.

Several limitations of the study need to be acknowledged. First, although schools in the Appalachian region of three states were included in the study, these findings may not be representative of youth perspectives in the full 13-state Appalachian region. Also, only public schools were included in the sample; thus, it is possible that students attending private schools may have different tobacco experiences or views. Second, responses were self-reported and thus subject to the potential of associated biases. Third, missing data (11.3%) may have influenced the results. Despite these limitations, however, study findings shed light on tobacco views and experiences of youth living in rural Appalachia.

This study contributes to the literature by providing substantial evidence that particular groups of youth tobacco users (e.g., e-cigarette only, poly) are more inclined to disagree with tobacco product harm statements (e.g., e-cigarettes). In particular, the study offers insights on the relationships between patterns of tobacco product use and harm perceptions among a vulnerable population of Appalachian youth.

## 5 Conclusion

In summary, although many youth participants had not tried tobacco or do not currently use tobacco products, the findings raise concern about the number of current conventional tobacco and e-cigarette users, especially polytobacco users. Most youth were aware of health dangers associated with smoking, but perceptions were split on whether e-cigarettes were associated with health problems or addiction. Tobacco use history appears to influence harm perceptions. Cigarette only users, polytobacco users, and e-cigarette only users were less likely to indicate that e-cigarettes cause health problems or addiction than never users. Taken as a whole, the findings of this study indicate the need for additional tobacco use prevention efforts with youth. In particular, campaigns addressing youth most vulnerable to try tobacco products or to continue use are needed.

## Funding

Research reported in this publication was supported, in part, by the National Heart, Lung, and Blood Institute (NHLBI) of the National Institutes of Health (NIH) and FDA Center for Tobacco Products under Award Numbers P50HL120163 and U54HL120163. The content is solely the responsibility of the authors and does not necessarily represent the official views of the NIH, the Food and Drug Administration, or the American Heart Association. The funding sponsors had no role in study design; data collection, analyses, or interpretation; manuscript preparation; or the decision to publish the results.

## CRediT authorship contribution statement

**Delvon T. Mattingly:** Conceptualization, Methodology, Software, Validation, Formal analysis, Writing - original draft, Writing - review & editing, Visualization. **Lindsay K. Tompkins:** Conceptualization, Methodology, Validation, Formal analysis, Writing - original draft, Writing - review & editing, Visualization. **Jayesh Rai:** Conceptualization, Methodology, Software, Formal analysis, Writing - review & editing, Visualization. **Clara G. Sears:** Conceptualization, Validation, Investigation, Resources, Data curation, Writing - review & editing. **Kandi L. Walker:** Conceptualization, Methodology, Validation, Investigation, Resources, Data curation, Writing - review & editing, Supervision, Project administration. **Joy L. Hart:** Conceptualization, Methodology, Validation, Investigation, Resources, Data curation, Writing - original draft, Writing - review & editing, Visualization, Supervision, Project administration, Funding acquisition.
